# Double coin in esophagus at same location and same alignment - a rare occurrence: a case report

**DOI:** 10.4076/1757-1626-2-7758

**Published:** 2009-07-16

**Authors:** Eti V Upadhyaya, Punit Srivastava, Vijay D Upadhyaya, AN Gangopadhyay, SP Sharma, DK Gupta, Zaheer Hassan

**Affiliations:** 1 Department of ENT, IMS, BHUVaranasi - 221005India; 2 Department of Pediatric Surgery IMS, BHUVaranasi - 221005India

## Abstract

Coin is the most common foreign body swallowed by pediatric age group. The multiple coin swallowing is extremely rare and very few cases had been reported in English literature. Most of them were present at different site and had different alignment in the esophagus. The location of the coin (trachea vs. esophagus) is commonly determined by the alignment of the coin on radiographic studies. A 4-year-girl was presented to us with history of coin ingestions one day back without any respiratory distress. On radiological study there was suspicion of two coins on same location and alignment. The diagnosis was confirmed after removal. The both coin was removed successfully by esophagoscopy. Unexpected second foreign bodies in pediatric esophageal coin ingestions are rare and it is mandatory to do post operative radiography after removal to exclude duplex coin or tracheal coin. We are presenting this case because of its rarity, difficulty in diagnosis especially when proper history is not available.

## Introduction

Ingestion of foreign bodies into the esophagus is encountered frequently in children and presents as an acute emergency in clinical practice. Coins, piece of toys etc. are some of the foreign bodies commonly seen [1]. The commonest FB that are impacted in the esophagus are coins [2,3]. A foreign body once ingested causes variable degree of esophageal obstruction and rarely respiratory distress, especially in infants and children below three years of age [4] and most of the foreign bodies lodge at the level of cricophryanx [2]. Anteroposterior (AP) and lateral chest roentgenograms can confirm the diagnosis of a coin in the esophagus. Esophageal coins align so as to appear as a circular disc (en face) on the AP view and as a thick line (on edge) on the lateral view [5]. The purpose of reporting this doubtful situation when the both coins align each other at same location, it is difficult to judge the number of coins on x-ray and removal of coin just by Magil Forcep or by Foley catheter may miss the coin if they are multiple [6]. It was confirmed by esophagoscopy and gently removal at same time to avoid complication and missing other one.

## Case presentation

**Figure 1.**
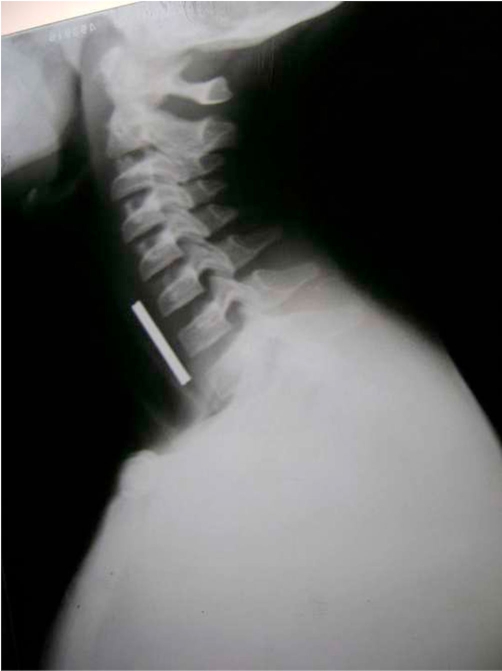
A.P. View X-ray chest showing coin in upper esophagus.

**Figure 2.**
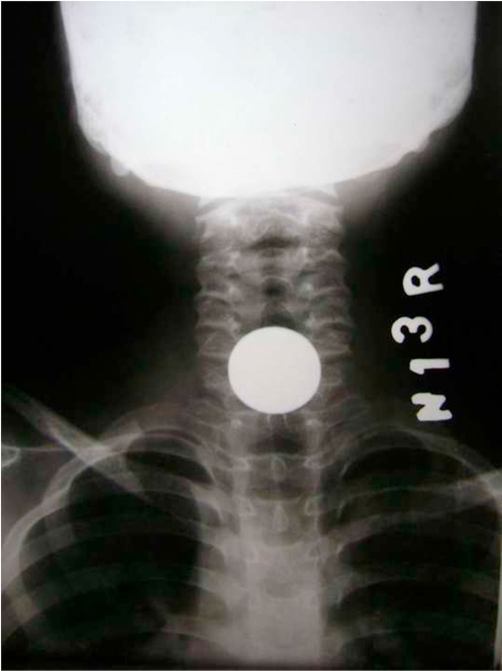
Lateral view X-ray showing double coin in upper esophagus.

**Figure 3.**
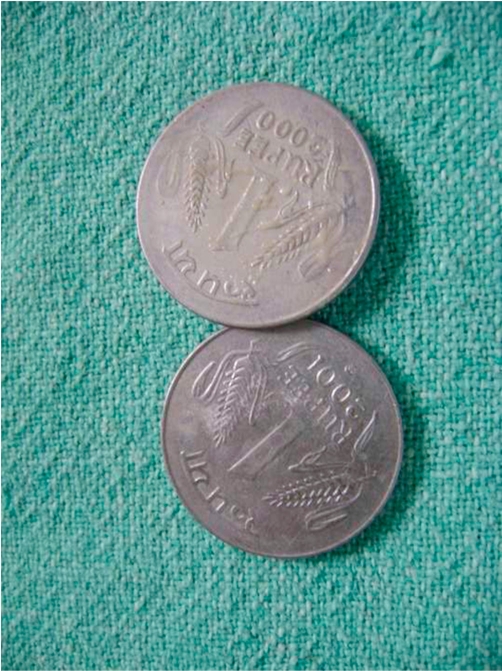
Two coins after removal.

A 4-year-old Indian girl was admitted to our hospital with history of coin ingestion accidentally a day before admission. The history was given by family member but not sure about the number of coin ingestion. On examination, child look was normal and there was no history of respiratory distress or cough. Roentgenogram anteroposterior view showed a coin sized round shadow a little above the third constriction of the esophagus (Figure 1). On closer view this round shadow looked slightly elongated longitudinally with some fading at the upper and lower edges, this was suggestive of the possibility of multiple coins, but not sure about multiple coin. On lateral view (Figure 2) there was increase thickness and groove in the radio-opaque shadow. The patient was planned for elective endoscopic extraction of foreign body (the coin) under general anesthesia. On esophagoscopy there were two coins which were stick to each other and lodged at cricopharynx. Both the coins (Figure 3) were removed successfully by grasping forceps without any trauma.

## Discussion

Though the coin is the commonest foreign body of esophagus in children but the occurrence of multiple coins is rather rare. Coins are lodged in upper esophagus immediately below the cricopharyngeus muscle in the coronal plane.[7] Once beyond the esophagus, coins often spontaneously pass unless other gastrointestinal pathology is present.[7] Removal of coins impacted in the esophagus is necessary because retained esophageal coins are associated with many complications. Various techniques have been described to deal with coins that lodge in the upper end of the esophagus including; rigid esophagoscopy [3], Foley’s catheter dislodgment [8], Magill’s forceps [9] and flexible esophagoscopy [10]. No clinician can fail to recognize the presence of a coin in the esophageal roentgenogram, but to delineate two objects, such as coins of the same size and shape is truly a challenge. These objects tend to stick together and the double dimensional projections often fail to indicate their presence, therefore it should be kept in mind that foreign body cases may be multiple, so proper history as well as close and careful evaluation of AP and lateral view of x-ray give clue to the diagnosis [6]. In our case both coins appear as a circular disc overlapped on each other in the AP view so it was not differentiated as two foreign bodies. Even on the lateral view we are not able to differentiate double thick line suggestive of two coins. So we suggest the extraction of coin should be done by endoscope when ever possible. In the places were endoscopic facilities are not available the repeat roentgenogram after extraction of coin is mandatory.
